# Italian Honeydew Honey Characterization by ^1^H NMR Spectroscopy

**DOI:** 10.3390/foods14132234

**Published:** 2025-06-25

**Authors:** Dalila Iannone, Laura Ruth Cagliani, Roberto Consonni

**Affiliations:** Institute of Chemical Sciences and Technologies “G. Natta” (SCITEC), National Research Council, Via Corti 12, 20133 Milan, Italy; dalila.iannone@scitec.cnr.it (D.I.); lauraruth.cagliani@scitec.cnr.it (L.R.C.)

**Keywords:** honeydew honey, NMR spectroscopy, botanical origin, multivariate statistical analysis

## Abstract

Honeydew honey represents a bee-derived product with different organoleptic characteristics and distinct properties with respect to floral honey. The market interest in honeydew honey has been growing in recent years due to its higher bioactive characteristics with respect to floral honey. The need for a deeper chemical characterization aimed to evaluate a possible botanical differentiation attracted the use of different analytical approaches. The present work aims to distinguish the botanical honeydew origin by using Nuclear Magnetic Resonance (NMR) spectroscopy and a multivariate approach. Two different data pretreatments have been considered to obtain the best sample discrimination. The saccharide content significantly affects the differentiation of the botanical variety consisting of fir, oak, citrus fruits, eucalyptus, and forest mainly by using a classification approach taking advantage of the Orthogonal Signal Correction filters. Notwithstanding the botanical diversity of the honeydew honey (HDH) samples, fir honeydew (F-HDH), oak honeydew (O-HDH), and eucalyptus honeydew (E-HDH) resulted always well discriminated among all the botanical varieties investigated, while citrus fruits honeydew (CF-HD) and forest honeydew (FO-HDH) did not. In particular, F-HDH resulted characterized by sucrose, erlose, maltose, maltotriose, maltotetraose, and melezitose, E-HDH resulted enriched in α, β-glucose and β-fructose in furanosidic form, and O-HDH enriched in β-fructose in furanosidic form, isomaltose.

## 1. Introduction

Honeydew honey (HDH) is produced by bees (*Apis mellifera*) foraging and collecting secretions produced by aphids, which draw their nourishment directly from the lymph of certain plants. Honeydew is collected by honeybees in the absence of floral sources of nectar, and it is processed with the addition of further enzymes into a dark-colored and strongly flavored specialty honey. According to entomological signatures in honey based on DNA metabarcoding of plant-sucking insects in agricultural and forest landscapes [1], forest honeydew honey mostly derived from the aphid *Cinara pectinatae*, while other botanical origins seem derived from the planthopper *Metcalfa pruinosa*. HDH is produced in different areas worldwide, mostly the United States, central/eastern Europe, and New Zealand, where its importance has increased as modern agricultural practices reduce available floral sources of nectar [2,3]. The organoleptic characteristics of HDH are very different from the floral honey, and HDHs are known for their higher biological activities, such as strong antioxidant and anti-inflammatory effects [4,5,6,7]. In addition, HDH shows higher pH values, higher conductivity, mineral content, and appreciably higher oligosaccharide levels than floral honey [8]. Notwithstanding, the chemical composition is still poorly characterized, either in relation to the production areas or the botanical differentiation.

Only in recent years, given the growing interest in honeydew honey, especially for its superior health-beneficial properties, have researchers devoted more efforts to the characterization of HDH. The different botanical origins affect the chemical composition and, consequently, the quality, aroma, and nutritional properties of the products: this strongly motivated the chemical investigation with different analytical approaches.

Actually, recent studies suggested a possible differentiation between floral honey and HDH, based on saccharides content [9], consisting of the latter, in general, lower amount of glucose and fructose, while conversely showed a higher amount of melezitose, erlose, and gluconic acid. In addition, several cyclic compounds (cyclitols) have been suggested to play the role of a marker for specific botanical origin of floral honey [10] or HDH [11,12].

The traditional chemical–physical parameters, such as optic rotation, major sugars, melissopalynological information, ash, pH, etc., are still the predominant characterization of bee products, including HDH, even though not as comprehensive as that for the floral one. In this respect, among advanced techniques, NMR represents a well-established powerful tool in aiming either chemical characterization or sample differentiation with the aid of multivariate analysis (MVA) strategies. Previous works have already highlighted the capability of NMR in honey characterization [13,14,15,16], and the aim of the present study is to address the botanical differentiation of Italian honeydew honey and to elucidate possible markers of honeydew according to the botany by using NMR spectroscopy and MVA.

## 2. Materials and Methods

A total of 48 honeydew honey (HDH) samples, consisting of 5 citrus fruits honeydew (CF-HDH), 10 eucalyptus honeydew (E-HDH), 6 fir honeydew (F-HDH), 21 forest honeydew (FO-HDH), and 6 oak honeydew (O-HDH) of different Italian territories were investigated, details reported in Table 1. 100 mg of HDH were dissolved at room temperature (25 °C) in vials by using 600 μL of deuterated water buffered at pH 7.54 (D_2_O, Merck, 99.96 atom % D, Milan, Italy) and 50 μL of TSP solution (Sodium Trimethylsilyl Propionate 13.35 mM in D_2_O). Samples were vortexed for 1 min and left overnight at 5 °C in the dark before NMR analysis.

### 2.1. NMR Data Acquisition and Processing

All NMR spectra were recorded on Bruker AVANCE NEO 600 spectrometer (Bruker Biospin, GmbH Rheinstetten, Karlsruhe, Germany, with Bruker Bio-spin software TOPSPIN 4.1.4 version), operating at 14.07 T, equipped with a 5 mm reverse Z gradient cryoprobe Prodigy, and thermostated autosampler, at 300 K. One-dimensional modified Nuclear Overhauser Effect Spectroscopy sequence (NOESY) was employed in a quantitative mode, including a solvent presaturation scheme with 128 scans over 64K of data, and a spectral width of 12 ppm, while a total relaxation time of 30 s was used to allow the complete proton relaxation. A zero filling to 128K and a resolution enhancement function (line broadening of 0.2 Hz) were applied before the Fourier transformation. All spectra were phased, automatically baseline corrected, and aligned with respect to α-glucose anomeric proton occurring at 5.23 ppm and 94.95 ppm for ^1^H and ^13^C, respectively. After the exclusion of residual solvent signal between 4.74 and 4.80 ppm, spectra were subjected to fixed bucketing (size of 0.02 ppm) in the range of 0.50–10.00 ppm. For each spectrum, the bucket normalization was performed with respect to the value of the total integral using the ACD/Spec Manager (ACD Labs, version 11, Toronto, ON, Canada). Bidimensional ^1^H-^13^C Heteronuclear Single Quantum Coherence (HSQC) and ^1^H-^1^H Total Correlation Spectroscopy (TOCSY) spectra have been recorded with the same spectral width parameters of the monodimensional spectra. Resonance assignment has been performed by using ^1^H NMR spectra of reference compounds, bidimensional heteronuclear experiments, and an available database repository (https://www.hmdb.org (accessed on 7 May 2025); CHENOMX NMR suite V12.0; https://bmrb.io/metabolomics (accessed on 7 May 2025)).

The quantification of 5-(hydroxymethyl)furfural (HMF) was carried out by adopting the TSP signal as an internal reference by using the following equation [17]:

$$\frac{Mx}{My}=\frac{Ix}{Iy}*\;\frac{Ny}{Nx}$$

where *M*, *I*, and *N* indicate molarity, signal integral value, and the number of protons generating the selected integrated signals for HMF (X) and for the reference standard used, TSP (Y).

### 2.2. Statistical Methods

NMR data were imported into SIMCA-P 18.0.1 (Sartorius Data Analytics, Umeå, Sweden) for multivariate statistical analysis. Principal Component Analysis (PCA) Projection to Latent Structures Discriminant Analysis (PLS-DA) was performed by using both “Mean Centering” and “Unit Variance” as data pretreatment. To overcome the randomness of PLS-DA models, the permutation test was checked. One Orthogonal Signal Correction (OSC) filter [18] has also been applied as data pretreatment to all PLS-DA models.

## 3. Results and Discussion

**Figure 1:**
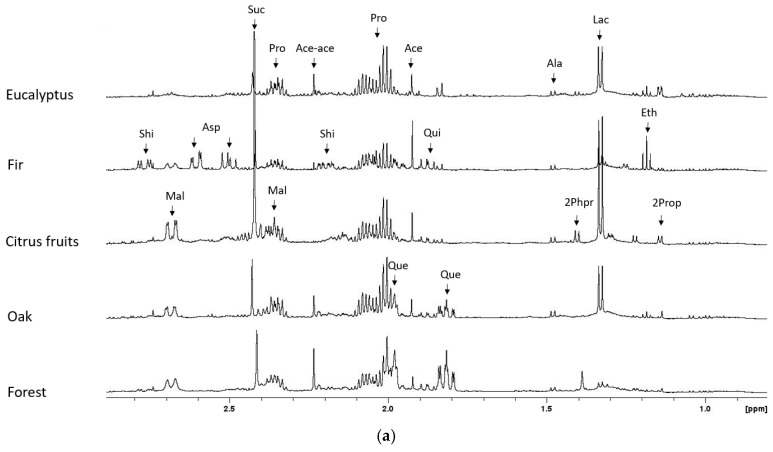

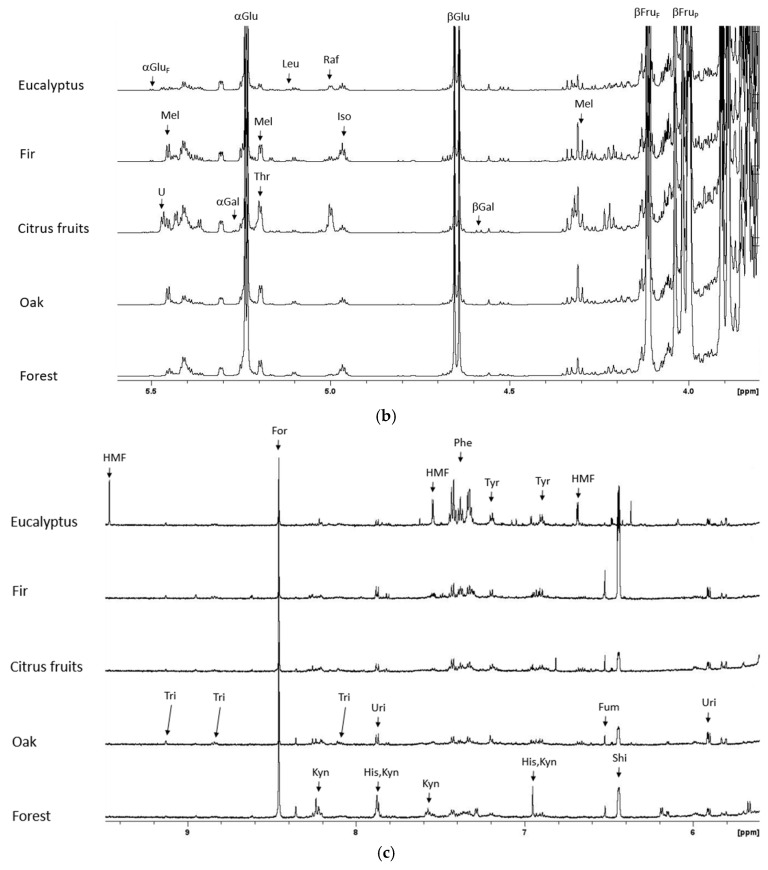
Selected ^1^H NMR spectral (**a**) aliphatic, (**b**) anomeric, and (**c**) aromatic regions of the HDH samples. Acronyms stand for: Ace, acetate; Ace-ace, acetoacetate; Ala, alanine; Asp, aspartate derivative; Eth, ethanol; For, formate; βFru_F_, β-fructose in furanosidic form; βFru_P_, β-fructose in pyranosidic form; Fum, fumarate; αGal, α-galactose; βGal, β-galactose; αGlu, α-glucose; βGlu, β-glucose; αGlu_F_, α-glucofuranose; His, histidine; HMF, 5-(hydroxymethyl)furfural; Iso, isomaltose; 2Prop, 2-propanol; Kyn, kynurenic acid; Lac, lactate; Leu, leucrose; Mal, malate; Mel, melezitose; Phe, phenylalanine; 2Phpr, 2-phenylpropanol; Pro, proline; Que, quercitol; Qui, quinic acid; Raf, raffinose; Shi, shikimate; Suc, succinate; Thr, threalose; Tri, Trigonelline; Tyr, tyrosine; U, unknown; Uri, uridine.

The analysis of all HDH samples allowed us to highlight those showing the most shared metabolite content within each botany. A representative ^1^H NMR spectrum of each single HDH botany was selected and depicted in Figure 1, including details of the resonances assignment. The collection of overlapped spectra for all botanical origins is included in Figure S1.

### 3.1. Aliphatic Spectral Region

The aliphatic region represented in Figure 1a showed the presence of a few characteristic amino acids, already observed in floral honey, like proline (2.35 ppm, 2.08 ppm, and 2.01 ppm) and alanine (1.48 ppm), organic acids like lactate (1.33 ppm), succinate (2.42 ppm) particularly relevant in CF-HDH, shikimate (2.77 and 2.20 ppm), quinic acid (1.97 and 1.88 ppm), malate (2.68 and 2.37 ppm), while acetate (1.92 ppm), acetoacetate (2.23 ppm), and ethanol (1.18 ppm) were observed in only few samples, most likely due to fermentation processes. In this respect, other fermentation products have been observed, like 2-propanol (1.14 ppm) and 2-phenylpropanol (1.40 ppm) in higher amounts in CF-HDH. Among all the metabolite patterns detected in ^1^H NMR spectra, selected metabolites appear to be potential indicators of the botanic classification. In detail, aspartate derivative (2.61 ppm and 2.50 ppm) was detected only in F-HDH, a large amount of 5-deoxyinositol (quercitol, 1.98 ppm and 1.82 ppm) was present in O-HDH and FO-HDH while shikimate was observed in high amount in F-HDH but also present in CF-HDH, O-HDH, and FO-HDH. This last botany did not show specific botanical markers, appearing enriched in a pool of metabolites observed in all the other HDH. Shikimic acid and its derivatives are quite usual in several honey and HDH varieties, being a key plant intermediate of the metabolic pathway of aromatic amino acids, particularly phenylalanine. It represents the main bridge between saccharide metabolism and secondary metabolism. High acetic acid content was already reported in HDH [19], although its formation by microbial metabolism is still not disclosed. Also, quercitol was found as a characteristic compound and suggested as a potential marker of O-HDH [9,20]. Quercitol, like other cyclitols, is likely to contribute to health-beneficial properties with an antiradical activity [21].

### 3.2. Anomeric Spectral Region

**Figure 2:**
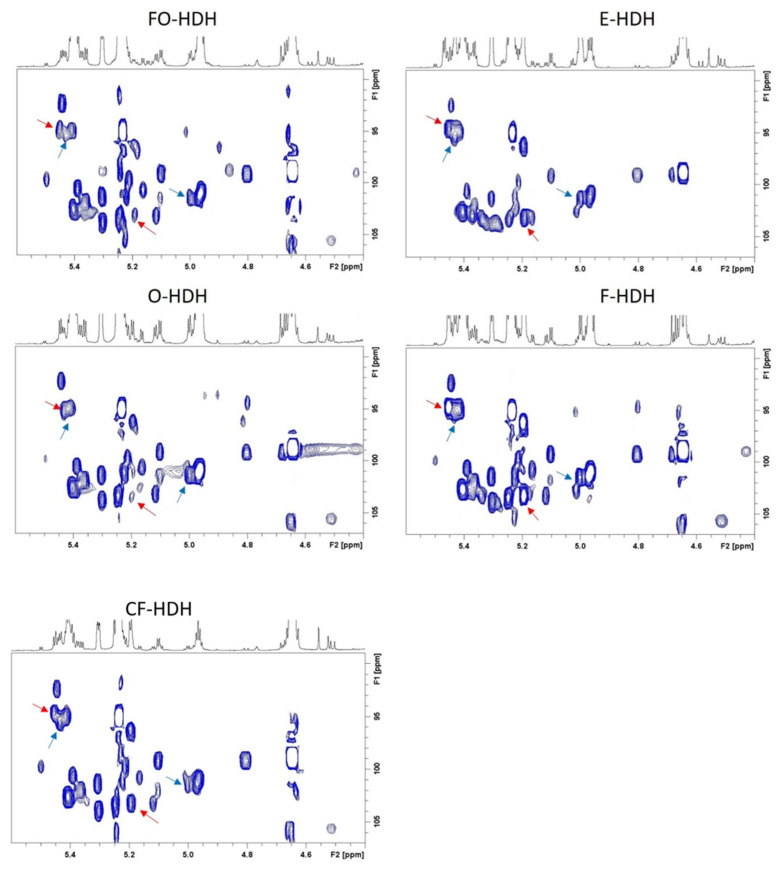
Anomeric spectral regions for ^1^H-^13^C HSQC spectra of HDH samples. In particular, red and blue arrows indicate melezitose and raffinose anomeric signals, respectively.

The most important spectral region is the so-called “anomeric region”, represented in Figure 1b, which includes the anomeric protons of all the saccharides, spanning mono- up to tetrasaccharides, and most of them already determined in floral honey [15]. In comparison with floral honey, all HDH samples appear to contain the same saccharides moieties with a larger amount of the trisaccharide melezitose (anomeric protons at 5.45 ppm, 5.20 ppm, and 4.30 ppm), the disaccharide leucrose (anomeric proton at 5.12 ppm), and the disaccharide isomaltose (anomeric protons at 5.25 ppm, 4.96 ppm, and 4.68 ppm). Melezitose was largely present in F-HDH and O-HDH, while the trisaccharide raffinose (anomeric protons at 5.43 ppm, 5.00 ppm, and 4.23 ppm) and the disaccharide threalose (anomeric proton at 5.20 ppm), observed in all HDH were largely present in CF-HDH, and isomaltose was found to be abundant in F-HDH, supporting the proposal that raffinose could play a role in botanical differentiation, according to previous determinations [22]. Interestingly, NMR signals of α-glucose in furanosidic form, present in all HDH and in floral honey, were observed (anomeric ^1^H at 5.50 ppm and ^13^C at 99.50 ppm) along with an unknown saccharide (anomeric proton at 5.47 ppm, and ^13^C at 94.67 ppm) present only in E-HDH and CF-HDH, and never observed in floral honey, most likely due to a phosphorylated saccharide moiety. In addition, α and β anomeric protons of galactose were detected at 5.27 ppm and 4.59 ppm, respectively, and never observed in floral honey. Signals of kojibiose (anomeric protons at 5.44 ppm, 5.39 ppm, and 5.10 ppm) and signal at 5.41 ppm, including overlapped saccharides (sucrose, erlose, maltose, maltotriose, maltotetraose) were identified. The complete content of saccharide content is represented in the ^1^H-^13^C HSQC spectra of Figure 2, with the inclusion of the additionally identified signals of melezitose and raffinose.

### 3.3. Aromatic Spectral Region

Interestingly, the aromatic spectral region represented in Figure 1c showed the presence of common compounds like shikimate (6.44 ppm), particularly relevant in F-HDH, while in a lower amount in CF-HDH, O-HDH, FO-HDH and absent in E-HDH, while kynurenic acid (8.23 ppm, 7.87 ppm, 7.57 ppm and 6.95 ppm) was only present in FO-HDH. In particular, kynurate is a tryptophan metabolite, a byproduct of the kynurenine metabolic pathway. It has been detected in several fields, ranging from honeybee products, plants, herbs, and spices to cells and human and animal tissues, showing anti-convulsant and neuroprotective activity. It is a common metabolite in chestnut honey [23,24]. Other metabolites, including organic acids and amino acids like fumarate (6.52 ppm), formate (8.46 ppm), phenylalanine (7.42 ppm, 7.37 ppm and 7.32 ppm), tyrosine (7.20 ppm and 6.90 ppm), histidine (7.88 ppm and 6.96 ppm) and nucleotides like uridine (7.88 ppm and 5.91 ppm), were also present in different amount according to the botanical origin, and already detected in floral honey samples. Interestingly, in all HDH, the pyridinic alkaloid trigonelline (9.13 ppm, 8.84 ppm, and 8.08 ppm) was also detected, even though in a very low amount. Trigonelline, a pyridine alkaloid, is the main alkaloid component of legume fenugreek [25] and was detected in specific floral botany like citrus and coffee honey [26,27]. Its biological activity includes hypoglycemic, hypolipidemic, and neuroprotective actions; it is generally involved in plant growth and development, playing the role of a nutrient source. Notably, significant levels of HMF (9.46 ppm, 7.54 ppm, and 6.69 ppm) were observed only in a few samples of CF-HDH, FO-HDH, and E-HDH samples. The complete resonance assignment of the identified metabolites is summarized in Table S1.

### 3.4. Multivariate Statistical Analysis

The evaluation of possible botanical differentiation was achieved by applying the multivariate statistical treatment to NMR data. Firstly, OSC PCA models were investigated for all HDH samples using both data pretreatments to reduce the dimensionality of the NMR data while preserving most of the variability as well as underlying patterns and relationships between the samples. As represented in Figure S2, a partial sample grouping was observed, suggesting the need for further investigation using discriminant analysis and considering samples in pairs.

**Figure 3:**
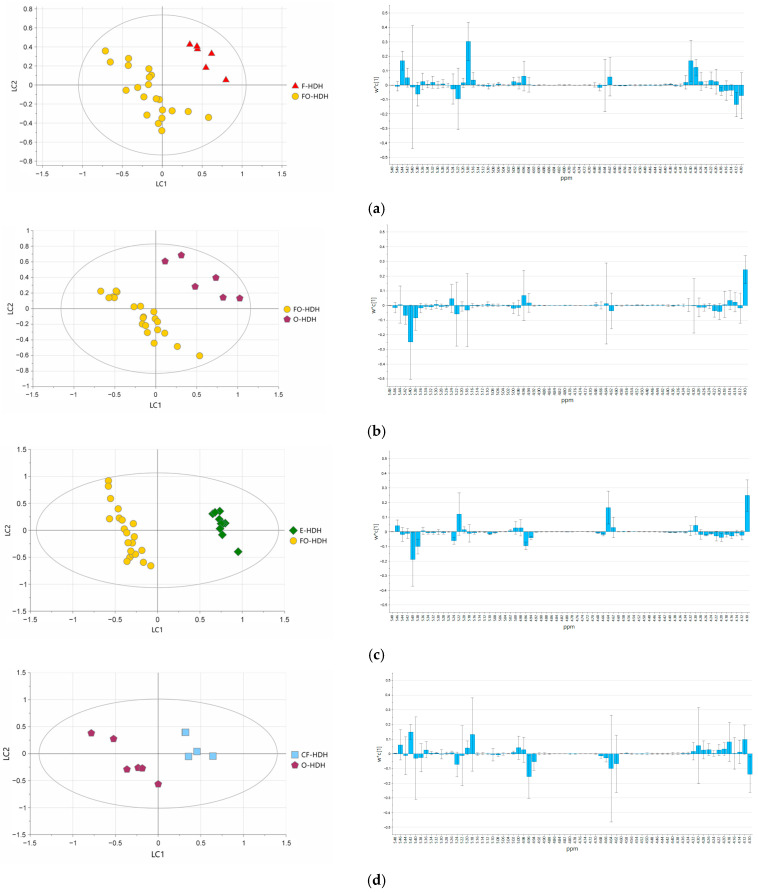

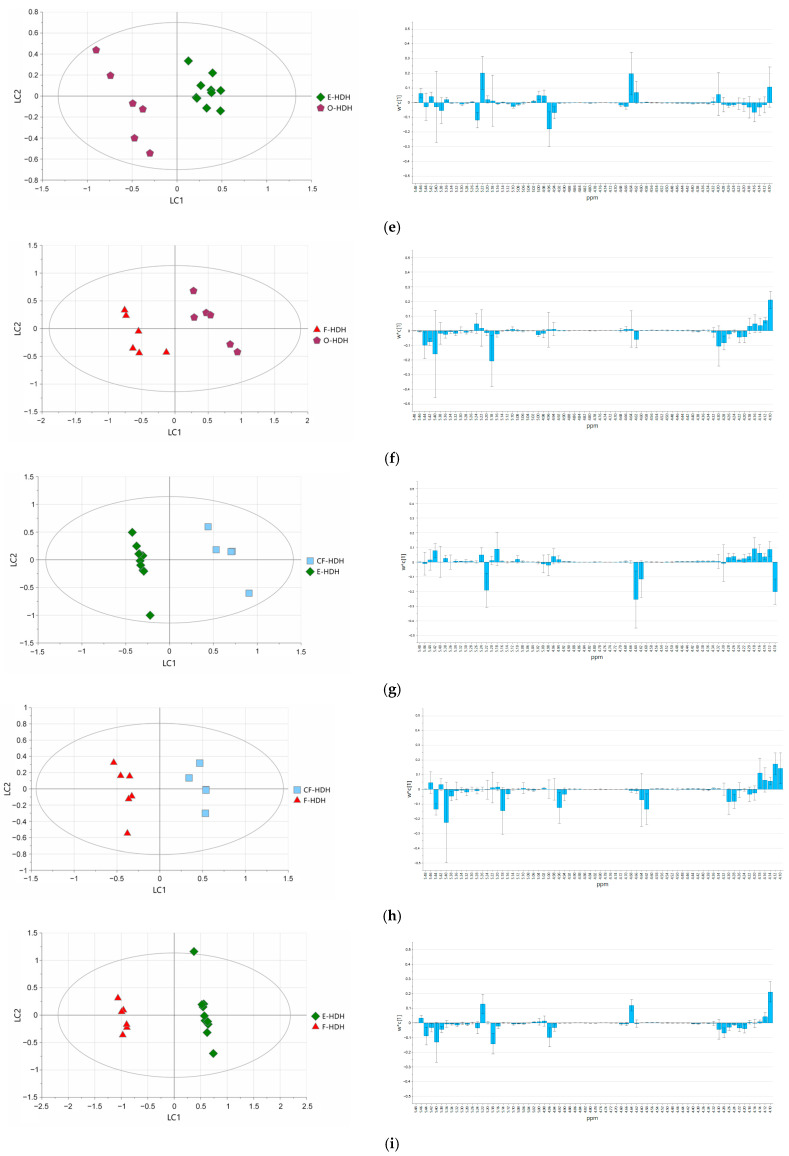
Score plot and the first component of loading plot (anomeric region) of two classes OSC PLS-DA models are represented on the left and right side, respectively, as following: (**a**) Fir and Forest honeydew (3LCs, LC1 = 28.7%, LC2 = 16.9%, R^2^X = 68%, R^2^Y = 96.4%, Q^2^ = 87.2%); (**b**) Forest and Oak honeydew (3LCs, LC1 = 33.9%, LC2 = 13%, R^2^X = 63.1%, R^2^Y = 97.4%, Q^2^ = 91.7%); (**c**) Eucalyptus and Forest honeydew (2LCs, LC1 = 33.9%, LC2 = 21.2%, R^2^X = 55%, R^2^Y = 98.8%, Q^2^ = 98%); (**d**) Citrus fruit and Oak honeydew (3LCs, LC1 = 34.1%, LC2 = 16.7%, R^2^X = 64.9%, R^2^Y = 97.5%, Q^2^ = 80.3%); (**e**) Eucalyptus and Oak honeydew (2LCs, LC1 = 34.8%, LC2 = 10.1%, R^2^X = 44.9%, R^2^Y = 95.6%, Q^2^ = 83.4%); (**f**) Fir and Oak honeydew (2LCs, LC1 = 48.4%, LC2 = 17.9%, R^2^X = 66.4%, R^2^Y = 95.9%, Q^2^ = 88.1%); (**g**) Citrus fruit and Eucalyptus honeydew (3LCs, LC1 = 44.4%, LC2 = 31.6%, R^2^X = 84.9%, R^2^Y = 99.7%, Q^2^ = 98.8%); (**h**) Citrus fruit and Fir honeydew (2LCs, LC1 = 38%, LC2 = 22%, R^2^X = 60%, R^2^Y = 98.1%, Q^2^ = 88.5%); (**i**) Eucalyptus and Fir honeydew (2LCs, LC1 = 64.1%, LC2 = 18%, R^2^X = 82.2%, R^2^Y = 99.9%, Q^2^ = 99.6%). Yellow dots, red triangles, purple pentagons, green diamonds, and light blues boxes stand for Forest, Fir, Oak, Eucalyptus, and Citrus fruit honeydew, respectively.

A single OSC filter has been applied to remove systematic variance in the dataset not related to the sample class (dummy variables) [28]. In order to evaluate the impact of distinct data pretreatment approaches on the performances of HDH differentiation models, two pre-processing steps have been prospected in the frame of the present study, considering the complete metabolite profiles of spectra. As a result, the adoption of “unit variance” treatment produced only five stable OSC PLS-DA models, allowing the discrimination between botanical species as follows: F/CF-HDH, F/E-HDH, CF/E-HDH, O/E-HDH, and O/CF-HDH. The “Unit Variance” data pretreatment mainly suffers the limited accessible evaluation of discriminant variables and additionally did not produce affordable models, most likely due to the intrinsic variability of the profiles in aliphatic and aromatic regions of the ^1^H NMR spectrum of HDH samples, where signals of less abundant compounds are fluctuating. These last-mentioned spectral regions highlighted the presence of minor components, like amino acids, organic acids, and nucleotides, in a low and variable amount. Conversely, by using “Mean Centering” data pretreatment, affordable OSC PLS-DA models were obtained for all botanical origin comparisons apart from only FO/CF-HDH. “Mean Centering” is commonly used to adjust the differences between low- and high-concentration metabolites by scaling all values so that they vary around zero. By using this data pretreatment, the intense signals were primarily considered, highlighting the relevance of saccharide content in sample discrimination. For this reason, only the anomeric spectral region has been considered for a precise marker evaluation, considering significant contributions for absolute w*c [1] values larger than 0,1. The loading plot of OSC PLS-DA models was presented in detail in Figure 3, along with the corresponding score plot (Figure S3 reports the OSC PCA models for samples in pairs). The loading plot of the F/FO-HDH model highlighted melezitose (buckets at 5.44 ppm, 5.18 ppm, 4.28 ppm, and 4.30 ppm) characterizing F-HDH while β-fructose in furanosidic form (bucket at 4.12 ppm) characterized FO-HDH (Figure 3a). The loading plot of the O/FO-HDH model indicated β-fructofuranose (bucket at 4.10 ppm) characterizing O-HDH while sucrose, erlose, maltose, maltotriose, and maltotetraose (bucket at 5.40 ppm) characterizing FO-HDH (Figure 3b).

The loading plot of E/FO-HDH model suggested sucrose, erlose, maltose, maltotriose, maltotetraose, kojibiose (buckets at 5.40 ppm and 5.38 ppm) characterizing FO-HDH while α, β-glucose and β-fructose in furanosidic form (buckets at 5.22 ppm, 4.64 ppm, and 4.10 ppm) were characterizing E-HDH (Figure 3c).

The loading plot of O/CF-HDH model indicated isomaltose, β-fructose in furanosidic form and β-glucose (buckets at 4.96 ppm, 4.64 ppm, and 4.10 ppm) characterizing O-HDH, while raffinose, melezitose and threalose (buckets at 5.42 ppm, and 5.18 ppm) were characteristic for CF-HDH (Figure 3d).

The loading plot of the E/O-HDH model indicated α, β-glucose and β-fructose in furanosidic form (buckets at 5.22 ppm, 4.64 ppm, and 4.10 ppm) characteristic for E-HDH while O-HDH was characterized by isomatose (buckets at 5.24 ppm, and 4.96 ppm) (Figure 3e).

The loading plot of O/F-HDH model indicated melezitose, sucrose, erlose, maltose, maltotriose, maltotetraose and threalose (buckets at 5.44 ppm, 5.40 ppm, 5.18 ppm, and 4.30 ppm) characterizing F-HDH while β-fructose in furanosidic form (bucket at 4.10 ppm) characterized O-HDH (Figure 3f).

The loading plot of the E/CF-HDH model indicated α, β-glucose, and β-fructose in furanosidic form (buckets at 5.22 ppm, 4.64 ppm, 4.62 ppm, and 4.10 ppm) characteristic for E-HDH (Figure 3g).

The loading plot CF/F-HDH model indicated melezitose, sucrose, erlose, maltose, maltotriose, maltotetraose, and threalose, and the open form of fructose and β-glucose (buckets at 5.44 ppm, 5.40 ppm, 5.18 ppm, 4.96 ppm, 4.62 ppm) characterizing F-HDH, while β-fructose in furanosidic form (buckets 4.12 ppm, 4.10 ppm) characterizing CF-HDH (Figure 3h).

Finally, the loading plot of the F/E-HDH model indicated sucrose, erlose, maltose, maltotriose, maltotetraose, melezitose, and threalose (buckets at 5.40 ppm, and 5.18 ppm) characterizing F-HDH while α, β-glucose, and β-fructose in furanosidic form (buckets at 5.22 ppm, 4.64 ppm, and 4.10 ppm) characterizing E-HDH (Figure 3i).

As previously mentioned, only FO/CF-HDH was not successfully discriminated, suggesting that the anomeric region did not significantly contribute to sample differentiation.

Chemical analysis of honeydew is not an easy task due to the high and different sugar content of the samples, which dominate the ^1^H NMR spectrum. It is known that honeydew carbohydrate composition varies among homopteran species and among host species [29,30], and its determination was investigated by different analytical techniques searching for possible markers of the authenticity of the botanical and/or geographical origin of honeydew honey. Recent studies [31,32] improved the chemical characterization of HDH from different origins by using mass spectrometry-based approaches, moving forward the knowledge on chemical composition and characterization of HDH samples. According to the present data, the use of NMR spectroscopy successfully allowed the differentiation of a botanical variety of Italian HDH samples, highlighting saccharide content as the main discriminant. The complete saccharide content determination is a very challenging task, which most likely could be attempted by a multi-analytical approach. Most traditional techniques, including chromatographic-based approaches, require sample derivatization, while the NMR technique does not. Still, the complex saccharide content disclosure of bee-based products is very challenging. Considering the botanical diversity of the HDH samples, it appears that F-HDH, O-HDH, and E-HDH always discriminated well among all the botanical varieties investigated, while CF-HD and FO-HDH did not. By considering the possible characteristic saccharides, resulting as relevant metabolites in OSC PLS-DA models, F-HDH appeared mostly characterized by the bucket at 5.40 ppm and 5.44 ppm, which contains the anomeric protons of sucrose, erlose, maltose, maltotriose, maltotetraose, and melezitose. E-HDH resulted clearly enriched in α, β-glucose, and β-fructose in furanosidic form with respect to all other botanical varieties, while O-HDH appears generally enriched in β-fructose in furanosidic form, isomaltose. The other two botanical varieties, CF-HDH and FO-HDH, revealed enrichment in different oligosaccharides according to pairs comparison as previously reported. Slight fluctuations are observed depending on the counterpart analyzed, but in general, the botanical origins appeared well characterized and differentiated. The presence of HMF, a furanic compound mainly produced by the saccharides degradation and considered a freshness indicator for honey [33], was evaluated in the samples investigated. Due to its harmful properties such as mutagenic, genotoxic, cytotoxic, and carcinogenic effects, according to the European Community COUNCIL DIRECTIVE 2001/110/EC [34], a limit level of HMF in honey was set to 40 mg kg^−1^, except for honey originating from the tropical region for which the limit was set at 80 mg kg^−1^. The NMR quantification performed on all HDH samples highlighted an over-the-limit amount of HMF in two CF-HDH, three FO-HDH samples, and one E-HDH sample, in full accordance with the previous determination (Table S2) [35]. The present work highlighted the possibility of differentiating Italian HDH based on the oligosaccharides content, allowing clear sample differentiation on the basis of NMR data and the use of OSC PLS-DA models. This result confirms the potentiality of the NMR approach in the characterization of saccharide mixtures, promoting its use in the authentication process. Interestingly, this was possible notwithstanding the metabolite variability of the samples within each botanical origin. Certainly, the possibility of enlarging the number of available samples would produce a more acute vision of the characteristic chemical composition of HDH samples, giving rise to better-defined statistical models, while the lack of HDH from another country, at the moment, impairs the possibility of a geographical origin discrimination.

## Figures and Tables

**Table 1:** List of honeydew samples analyzed with the indication of botany, geographical origin of Italian territory in which they were collected, and harvest year.

Sample	Botany	Geographical Origin	Harvest Year
1	Citrus fruits	Sardinia	2022
2	Citrus fruits	Sicily	2022
3	Citrus fruits	Veneto	2022
4	Citrus fruits	Sicily	2023
5	Citrus fruits	Sicily	2023
1	Eucalyptus	Basilicata	2022
2	Eucalyptus	Sardinia	2022
3	Eucalyptus	Sardinia	2022
4	Eucalyptus	Sardinia	2022
5	Eucalyptus	Sardinia	2022
6	Eucalyptus	Sardinia	2023
7	Eucalyptus	Sardinia	2023
8	Eucalyptus	Sardinia	2023
9	Eucalyptus	Sardinia	2023
10	Eucalyptus	Sardinia	2023
1	Fir	Abruzzo	2022
2	Fir	Trentino A. Adige	2022
3	Fir	Trentino A. Adige	2022
4	Fir	Tuscany	2022
5	Fir	Veneto	2022
6	Fir	Veneto	2022
1	Forest	Emilia Romagna	2022
2	Forest	Lombardy	2022
3	Forest	Lombardy	2022
4	Forest	Piedmont	2022
5	Forest	Trentino A. Adige	2022
6	Forest	Trentino A. Adige	2022
7	Forest	Tuscany	2022
8	Forest	Tuscany	2022
9	Forest	Veneto	2022
10	Forest	Veneto	2022
11	Forest	Calabria	2023
12	Forest	Campania	2023
13	Forest	Emilia Romagna	2023
14	Forest	Liguria	2023
15	Forest	Lombardy	2023
16	Forest	Lombardy	2023
17	Forest	Marche	2023
18	Forest	San Marino	2023
19	Forest	Tuscany	2023
20	Forest	Veneto	2023
21	Forest	Veneto	2023
1	Oak	Apulia	2022
2	Oak	Calabria	2022
3	Oak	Marche	2022
4	Oak	Trentino A. Adige	2022
5	Oak	Apulia	2023
6	Oak	Emilia Romagna	2023

## Data Availability

The original contributions presented in this study are included in the article/supplementary material. Further inquiries can be directed to the corresponding author.

## Supplementary Materials

The following supporting information can be downloaded at: https://www.mdpi.com/article/10.3390/foods14132234/s1, Figure S1: ^1^H NMR spectra expansion of aliphatic (a), anomeric (b), and aromatic (c) region of all overlapped honeydew samples analyzed; Figure S2: Score plot of OSC PCA models with two data pretreatments: (a) Mean Centering, (10PCs, PC1 = 30.2%, PC2 = 22%, R^2^X = 97.8%, Q^2^ = 85,2%) (b) Unit Variance, (9PCs, PC1 = 22.5%, PC2 = 10.8%, R^2^X = 74%, Q^2^ = 33%). Yellow dots, red triangles, purple pentagons, green diamonds, light blues boxes, stand for Forest, Fir, Oak, Eucalyptus, and Citrus fruit honeydew; Figure S3: Score plot of OSC PCA models as following: (a) Fir and Forest honeydew (4PCs, PC1 = 36.3%, PC2 = 26.1%, R^2^X = 87.1%, Q^2^ = 63.6%); (b) Forest and Oak honeydew (6PCs, PC1 = 38.7%, PC2 = 24.9%, R^2^X = 93.8%, Q^2^ = 77.3%); (c) Eucalyptus and Forest honeydew (9PCs, PC1 = 35%, PC2 = 25.8%, R^2^X = 98.3%, Q^2^ = 84.7%); (d) Citrus fruit and Oak honeydew (2PCs, PC1 = 37.1%, PC2 = 19.8%, R^2^X = 56.9%, Q^2^ = 3.32%); (e) Eucalyptus and Oak honeydew (2PCs, PC1 = 35.1%, PC2 = 32.8%, R^2^X = 67.9%, Q^2^ = 10.5%); (f) Fir and Oak honeydew (2PCs, PC1 = 49.6%, PC2 = 22.6%, R^2^X = 72.1%, Q^2^ = 37.3%); (g) Citrus fruit and Eucalyptus honeydew (6PCs, PC1 = 46.2%, PC2 = 30.9%, R^2^X = 98.1%, Q^2^ = 84.9%); (h) Citrus fruit and Fir honeydew (5PCs, PC1 = 38.1%, PC2 = 26.2%, R^2^X = 96.9%, Q^2^ = 80.1%); (i) Eucalyptus and Fir honeydew (4PCs, PC1 = 64.2%, PC2 = 19%, R^2^X = 96.5%, Q^2^ = 87.2%). Yellow dots, red triangles, purple pentagons, green diamonds, and light blues boxes stand for Forest, Fir, Oak, Eucalyptus, and Citrus fruit honeydew; Table S1: ^1^H NMR assignment of all compounds identified in honeydew samples; Table S2: Quantification of HMF for all honeydew samples analyzed.

## Abbreviations

The following abbreviations are used in this manuscript:

CF-HDH	Citrus fruit honeydew
E-HDH	Eucalyptus honeydew
F-HDH	Fir honeydew
FO-HDH	Forest honeydew
O-HDH	Oak honeydew
HDH	Honeydew honey
HMF	5-(hydroxymethyl)furfural
HSQC	Heteronuclear Single Quantum Coherence
MVA	Multivariate Analysis
NMR	Nuclear Magnetic Resonance
NOESY	Nuclear Overhauser Effect Spectroscopy
OSC	Orthogonal Signal Correction
PCA	Principal Component Analysis
PLS-DA	Projection to Latent Structures Discriminant Analysis
TOCSY	Total Correlation Spectroscopy
