# Supplementary material for: Italian Honeydew Honey Characterization by 1H NMR Spectroscopy
Source: Foods. 2025 Jun 25;14(13):2234. doi: 10.3390/foods14132234 (PMC12249152; doi:10.3390/foods14132234)
Supplement: Supplementary file 1 [file foods-14-02234-s001.zip › foods-3667432-supplementary.pdf]

# Supplementary Materials

## Special Issue: Application of NMR Spectroscopy in Food Analysis

# Italian honeydew honey characterization by $^{1}H$ NMR spectroscopy

**Dalila Iannone, Laura Ruth Cagliani and Roberto Consonni ***

National Research Council, Institute of Chemical Sciences and Technologies "G. Natta" (SCITEC), via Corti 12, 20133, Milan, Italy
* Correspondence: roberto.consonni@scitec.cnr.it; Tel.:+39 02 23699758

**Figure S1** $^{1}$H NMR spectra expansion of aliphatic (a), anomeric (b), and aromatic (c) region of all overlapped honeydew samples analyzed.

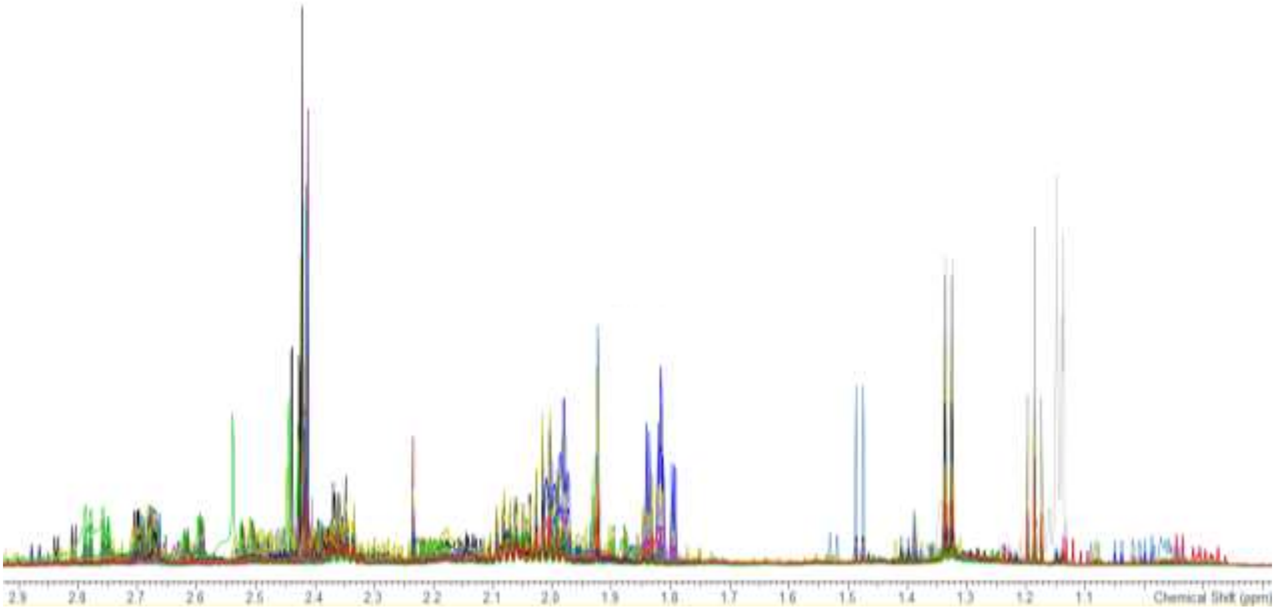


**(a)**

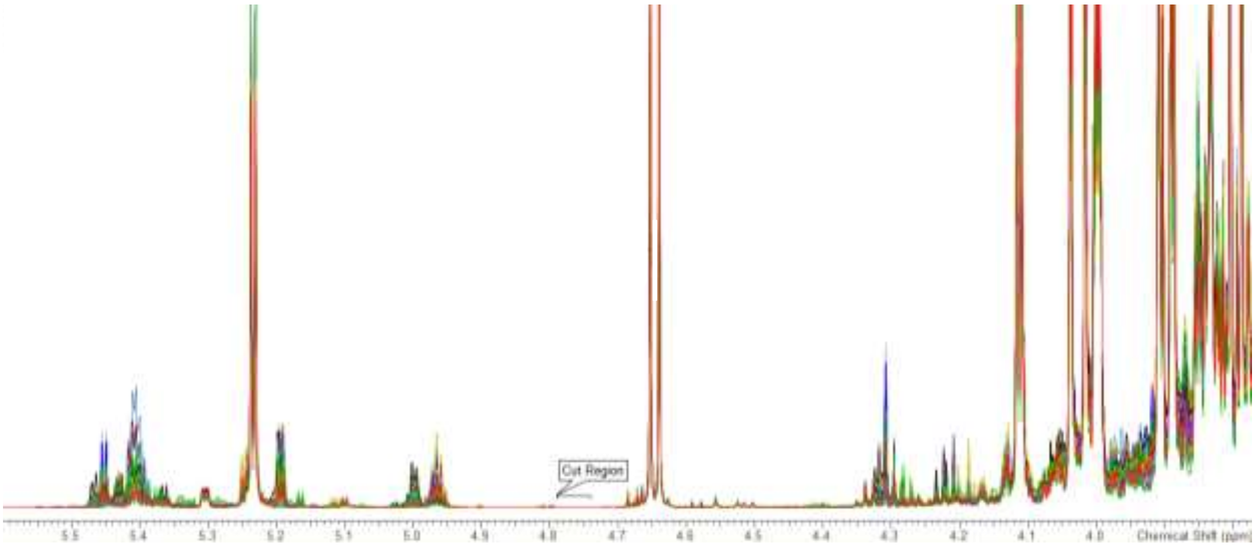


**(b)**

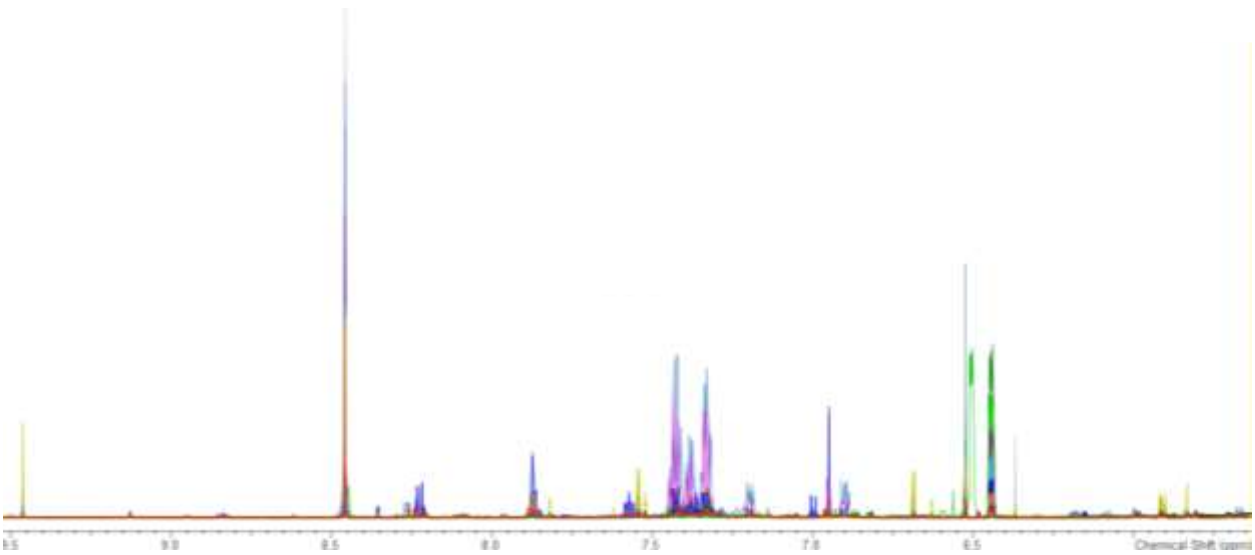


**(c)**

**Figure S2** Score plot of OSC PCA models with two data pre treatments: (a) Mean Centering, (10PCs, PC1=30,2%, PC2=22%, $R^2X$=97,8%, $Q^2$=85,2%) (b) Unit Variance, (9PCs, PC1=22,5%, PC2=10,8%, $R^2X$=74%, $Q^2$=33%). Yellow dots, red triangles, purple pentagons, green diamonds, light blues boxes, stand for Forest, Fir, Oak, Eucalyptus, and Citrus fruit honeydew.

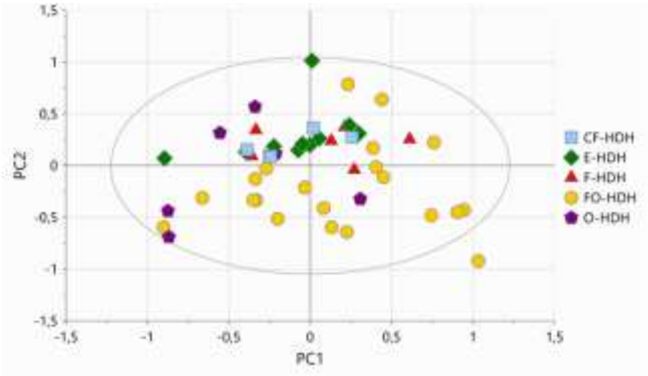


**(a)**

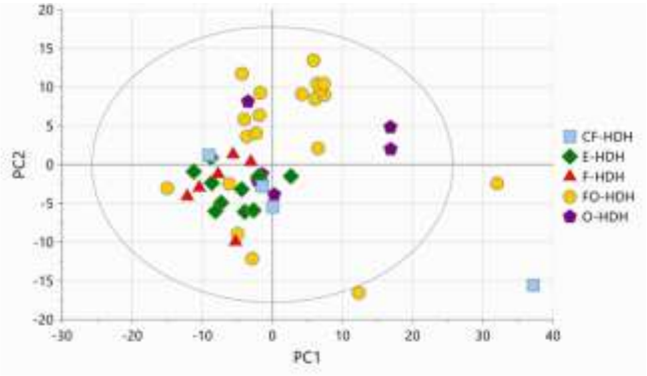


**(b)**

**Figure S3** Score plot of OSC PCA models, as following: (a) Fir and Forest honeydew (4PCs, PC1=36,3%, PC2=26,1%, $R^2X$=87,1%, $Q^2$=63,6%); (b) Forest and Oak honeydew (6PCs, PC1=38,7%, PC2=24,9%, $R^2X$=93,8%, $Q^2$=77,3%); (c) Eucalyptus and Forest honeydew (9PCs, PC1=35%, PC2=25,8%, $R^2X$=98,3%, $Q^2$=84,7%); (d) Citrus fruit and Oak honeydew (2PCs, PC1=37,1%, PC2=19,8%, $R^2X$=56,9%, $Q^2$=3.32%); (e) Eucalyptus and Oak honeydew (2PCs, PC1=35,1%, PC2=32,8%, $R^2X$=67,9%, $Q^2$=10,5%); (f) Fir and Oak honeydew (2PCs, PC1=49,6%, PC2=22,6%, $R^2X$=72,1%, $Q^2$=37,3%); (g) Citrus fruit and Eucalyptus honeydew (6PCs, PC1=46,2%, PC2=30,9%, $R^2X$=98,1%, $Q^2$=84,9%); (h) Citrus fruit and Fir honeydew (5PCs, PC1=38,1%, PC2=26,2%, $R^2X$=96,9%, $Q^2$=80,1%); (i) Eucalyptus and Fir honeydew (4PCs, PC1=64,2%, PC2=19%, $R^2X$=96,5%, $Q^2$=87,2%).Yellow dots, red triangles, purple pentagons, green diamonds, light blues boxes, stand for Forest, Fir, Oak, Eucalyptus, and Citrus fruit honeydew.

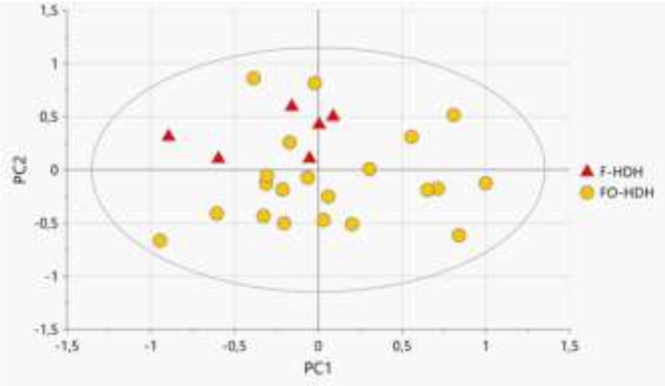


**(a)**

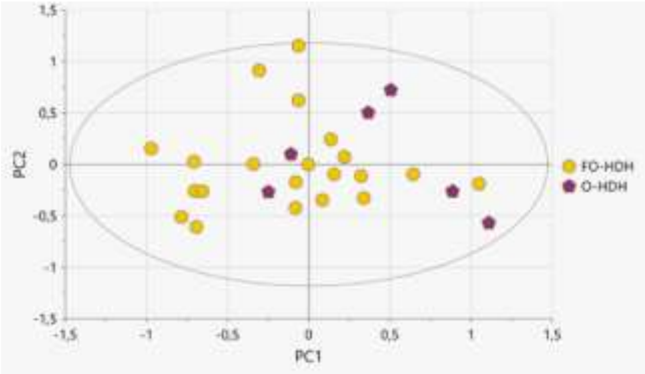


**(b)**

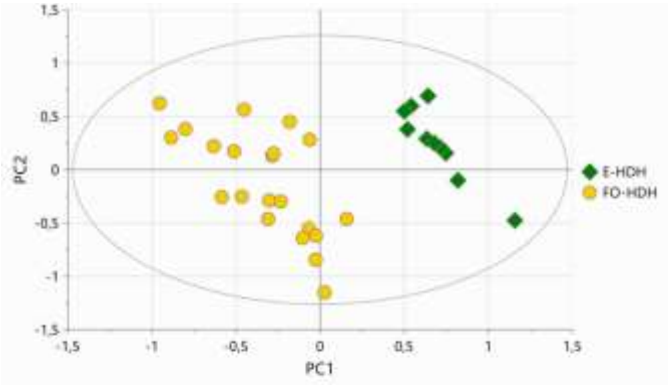


**(c)**

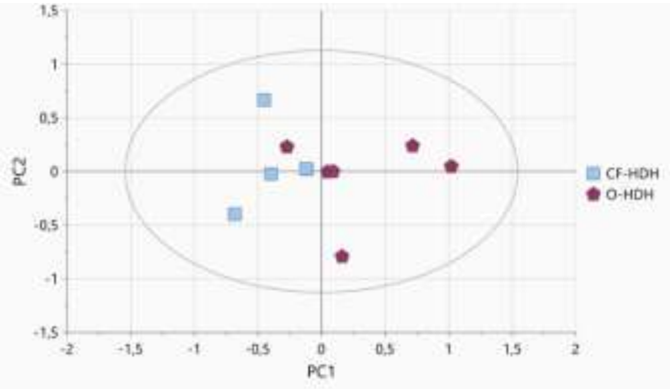


**(d)**

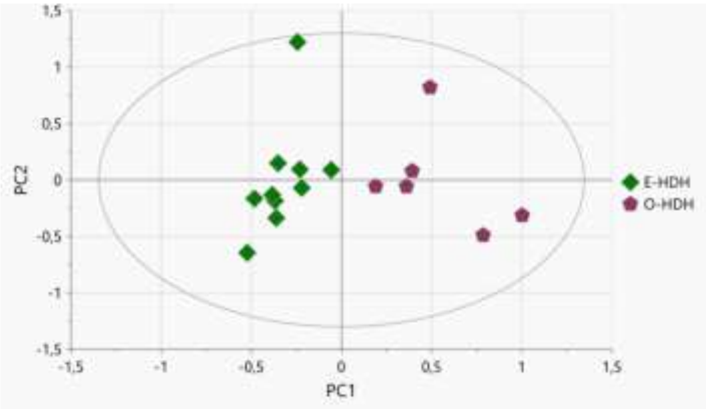


**(e)**

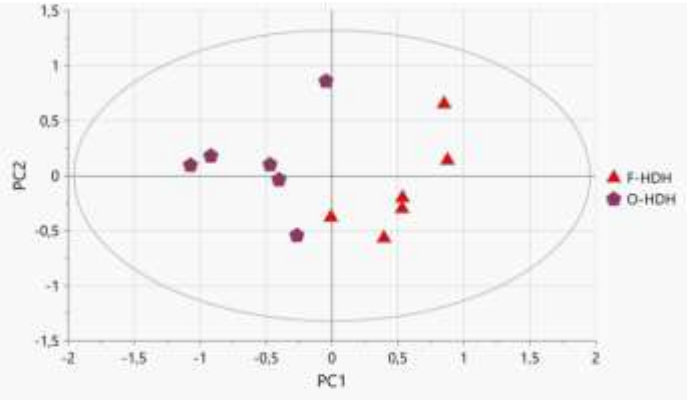


**(f)**

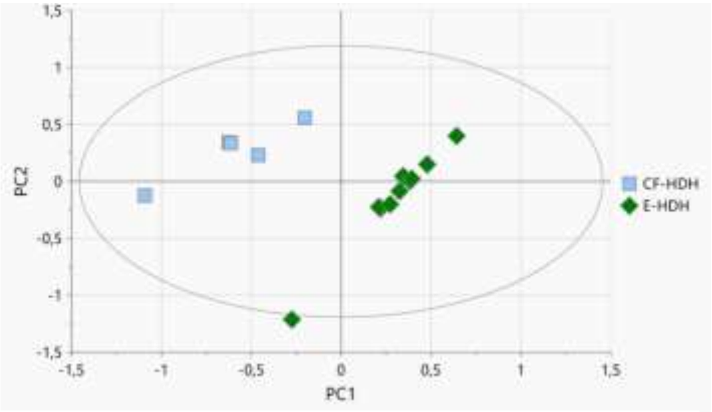
PC2
PC1
1,5
1
0,5
0
-0,5
-1
-1,5
-1,5
-1
-0,5
0
0,5
1
1,5
CF-HDH
E-HDH

**(g)**

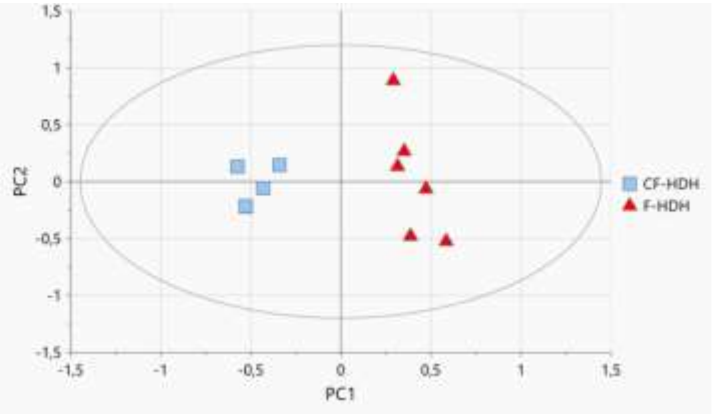
PC2
PC1
1,5
1
0,5
0
-0,5
-1
-1,5
-1,5
-1
-0,5
0
0,5
1
1,5
CF-HDH
F-HDH

**(h)**

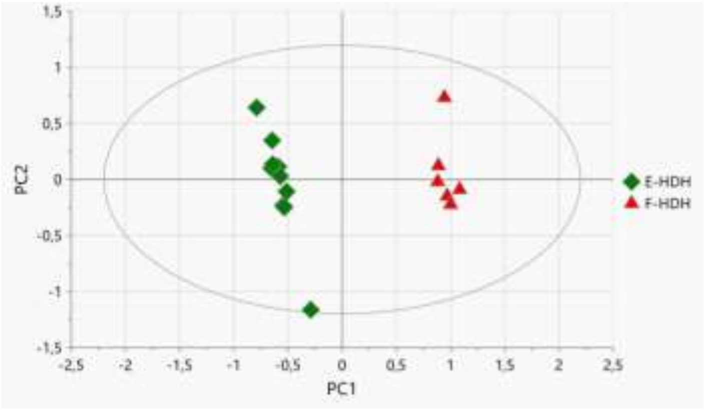
PC2
PC1
1,5
1
0,5
0
-0,5
-1
-1,5
-2,5
-2
-1,5
-1
-0,5
0
0,5
1
1,5
2
2,5
E-HDH
F-HDH

**(i)**

**Table S1** $^{1}H$ NMR assignment of all compounds identified in honeydew samples.

| Compound | $^{1}H$ chemical shift values (ppm) | | | |
|---|---|---|---|---|
| HMF | 9,46 | 7,54 | 6,69 | |
| Trigonelline | 9,13 | 8,84 | 8,08 | |
| Formate | 8,46 | | | |
| Kynurenic acid | 8,23 | 7,87 | 7,57 | 6,95 |
| Histidine | 7,88 | 6,96 | | |
| Uridine | 7,88 | 5,91 | | |
| Phenylalanine | 7,42 | 7,37 | 7,32 | |
| Tyrosine | 7,20 | 6,90 | | |
| Fumarate | 6,52 | | | |
| Shikimate | 6,44 | 2,77 | 2,20 | |
| aGlu$_F$ | 5,50 | | | |
| U | 5,47 | | | |
| Melezitose | 5,45 | 5,20 | 4,30 | |
| Kojibiose | 5,44 | 5,39 | 5,10 | |
| Raffinose | 5,43 | 5,00 | 4,23 | |
| Sucrose | 5,41 | | | |
| Erlose | 5,41 | | | |
| Maltose | 5,41 | 5,23 | 4,65 | |
| Maltotriose | 5,41 | 5,23 | 4,65 | |
| Maltotetraose | 5,41 | 5,23 | 4,65 | |
| aGal | 5,27 | | | |
| Isomaltose | 5,25 | 4,96 | 4,68 | |
| aGlu | 5,23 | | | |
| Threalose | 5,20 | | | |
| Leucrose | 5,12 | | | |
| bGlu | 4,65 | | | |
| bGal | 4,59 | | | |
| bFru$_F$ | 4,11 | | | |
| bFru$_P$ | 4,02 | | | |
| Malate | 2,68 | 2,37 | | |
| Aspartate derivative | 2,61 | 2,50 | | |
| Succinate | 2,42 | | | |
| Proline | 2,35 | 2,08 | 2,01 | |
| Acetacetate | 2,23 | | | |
| Quercitol | 1,98 | 1,82 | | |
| Quinic acid | 1,97 | 1,88 | | |
| Acetate | 1,92 | | | |
| Alanine | 1,48 | | | |
| 2-phenylpropanol | 1,40 | | | |
| Lactate | 1,33 | | | |
| Ethanol | 1,18 | | | |
| 2-propanol | 1,14 | | | |

**Table S2** Quantification of HMF for all honeydew samples analyzed.

| Sample | Botany | Geographical Origin | Harvest year | HMF (ppm) |
|---|---|---|---|---|
| 1 | Citrus fruits | Sardinia | 2022 | 153,28 |
| 2 | Citrus fruits | Sicily | 2022 | 22,76 |
| 3 | Citrus fruits | Veneto | 2022 | 51,33 |
| 4 | Citrus fruits | Sicily | 2023 | 33,23 |
| 5 | Citrus fruits | Sicily | 2023 | n.d. |
| 1 | Eucalyptus | Basilicata | 2022 | n.d. |
| 2 | Eucalyptus | Sardinia | 2022 | 47,64 |
| 3 | Eucalyptus | Sardinia | 2022 | 17,05 |
| 4 | Eucalyptus | Sardinia | 2022 | n.d. |
| 5 | Eucalyptus | Sardinia | 2022 | 5,67 |
| 6 | Eucalyptus | Sardinia | 2023 | n.d. |
| 7 | Eucalyptus | Sardinia | 2023 | 24,83 |
| 8 | Eucalyptus | Sardinia | 2023 | n.d. |
| 9 | Eucalyptus | Sardinia | 2023 | n.d. |
| 10 | Eucalyptus | Sardinia | 2023 | n.d. |
| 1 | Fir | Abruzzo | 2022 | 5,38 |
| 2 | Fir | Trentino A. Adige | 2022 | 18,21 |
| 3 | Fir | Trentino A. Adige | 2022 | n.d. |
| 4 | Fir | Tuscany | 2022 | 6,05 |
| 5 | Fir | Veneto | 2022 | n.d. |
| 6 | Fir | Veneto | 2022 | n.d. |
| 1 | Forest | Emilia Romagna | 2022 | n.d. |
| 2 | Forest | Lombardy | 2022 | 27,07 |
| 3 | Forest | Lombardy | 2022 | 100,78 |
| 4 | Forest | Piedmont | 2022 | n.d. |
| 5 | Forest | Trentino A. Adige | 2022 | n.d. |
| 6 | Forest | Trentino A. Adige | 2022 | 58,84 |
| 7 | Forest | Tuscany | 2022 | n.d. |
| 8 | Forest | Tuscany | 2022 | 15,93 |
| 9 | Forest | Veneto | 2022 | 54,32 |
| 10 | Forest | Veneto | 2022 | n.d. |
| 11 | Forest | Calabria | 2023 | 4,47 |
| 12 | Forest | Campania | 2023 | n.d. |
| 13 | Forest | Emilia Romagna | 2023 | n.d. |
| 14 | Forest | Liguria | 2023 | n.d. |
| 15 | Forest | Lombardy | 2023 | 34,38 |
| 16 | Forest | Lombardy | 2023 | n.d. |
| 17 | Forest | Marche | 2023 | n.d. |
| 18 | Forest | San Marino | 2023 | 29,43 |
| 19 | Forest | Tuscany | 2023 | 5,42 |

| | | | | |
|---|---|---|---|---|
| 20 | Forest | Veneto | 2023 | n.d. |
| 21 | Forest | Veneto | 2023 | n.d. |
| 1 | Oak | Apulia | 2022 | n.d. |
| 2 | Oak | Calabria | 2022 | 18,72 |
| 3 | Oak | Marche | 2022 | 7,43 |
| 4 | Oak | Trentino A. Adige | 2022 | 6,51 |
| 5 | Oak | Apulia | 2023 | 27,55 |
| 6 | Oak | Emilia Romagna | 2023 | n.d. |
